# Income-related children’s health inequality and health achievement in China

**DOI:** 10.1186/s12939-014-0102-6

**Published:** 2014-10-29

**Authors:** Lu Chen, Ya Wu, Peter C Coyte

**Affiliations:** Economics School, Nankai University, 94 Weijin Road, Nankai District, Tianjin, ON 300071 China; China Development Bank Heilongjiang Branch, 131 Xiangjiang Road, Heilongjiang, ON 150090 China; Institute of Health Policy, Management and Evaluation, University of Toronto, Health Sciences Building, 4th Floor, 155 College Street, Suite 425, Toronto, ON M5T3M6 Canada

**Keywords:** Child health inequality, Concentration index, Health achievement

## Abstract

**Introduction:**

This study assessed income–related health inequality and health achievement in children in China, and additionally, examined province-level variations in health achievement.

**Methods:**

Longitudinal data on 19,801 children under 18 years of age were derived from the China Health and Nutrition Survey. Income–related health inequality and health achievement were measured by the Health Concentration and Health Achievement Indices, respectively. Panel data with a fixed effect multiple regression model was employed to examine province-level variations in health achievement.

**Results:**

A growing trend was towards greater health inequality among Chinese children over the last two decades. Although health achievement was getting better over time, the pro-rich inequality component has lessened the associated gain in achievement. Health achievement was positively impacted by middle school enrollments, the urbanization rate, inflation-adjusted per capita gross domestic product, and per capita public health spending.

**Conclusion:**

This study has provided evidence that average health status of Chinese children has improved, but inequality has widened. Widening inequality slowed the growth in health achievement for children over time. There were wide variations in health achievement throughout China.

## Introduction

Since reforms started in 1978, China has experienced unprecedented economic growth. The economic boom has been accompanied by dramatic advances against extreme poverty. However, the benefits of growth are not distributed equally [1-3]. These inequalities in income between urban and rural areas, and between eastern and western regions have widened substantially [3,4]. Increasing income inequality may affect society and economic development in many ways [5]. In this paper, we focus on a particular aspect of health. Although health gains have continued, it is obvious that the evaluation of reform achievements need to take into account not only the average level of population health but also health inequalities [6]. The unequal distribution of health potentially adversely affects labor supply and productivity of lower income households, and consequently, may further exacerbate income inequality [7].

Children represent the future for society and are an important resource for sustainable development. Understanding the determinants of child health is important because health in childhood affects human capital accumulation and labor market status in adulthood [8-10]. Therefore, promoting children’s health and diminishing health inequality has become an important policy priority for the Chinese government.

A large portion of the existing literature mainly focusing on developed counties shows that family income is positively related to children’s general health in the United States [11,12], United Kingdom [13], Canada [14], Germany [15], and Australia [16]. However, little is known about the relationship between family income and child health in most developing countries. Wagstaff et al. [17] found inequalities in stunting among young children in Vietnam in both 1993 and 1998. Uthman [18] measured socio-economic inequality in Nigerian children under-five years of age using the extended concentration index and health achievement. Arokiasamy and Pradhan [19] explored income-related health inequality among Indian children. These studies consistently demonstrate a significant association between socio-economic status and child health outcomes.

Much of the prior literature on health inequalities in children in China has focused on health disparities between urban and rural areas. Shen et al. [20] used five sets of cross-sectional data between 1975 and 1992 and found that height differences between urban and rural children had increased since China’s Reform and Opening Policy. Luo et al. [21] examined the differences in height and body mass index for children in urban and rural areas in Hunan province and found that urban children were significantly taller and heavier than their rural counterparts in the 1990s and 2000s. Hejun and Yunping [22] examined income-related inequality in rural China using data from the 2004 and 2006 China Nutrition and Health Survey (CHNS) and found children of high-income families had better health status than those of low-income families. Chen et al. [7] and Liu et al. [23], using the 1989 to 2000 and 1989 to 2006 CHNS, respectively, analyzed urban–rural childhood malnutrition and supported the conclusion that inequality existed, but has been declining. All of the above literature related health inequality to individual (household)-level characteristics, such as age, gender, head’s education, household income, accessibility, rural–urban gap, and road accessibility. There has been a paucity of literature that assesses province-level health inequality and health achievement in Chinese children, thereby leaving them open questions. It is still unknown how health achievement for children is related to macro-level indicators (i.e. province’s economic performance, demographics and local government health spending). This paper aims to fill this gap in the international literature.

Our study will build on prior research by adding to the literature in two distinct ways. First, we will use the health concentration and health achievement indices to examine income–related health inequality and health achievement in children, respectively. Our study will use data from the last two decades, a period of unprecedented economic growth. In this study, the health of children will be measured in terms of height-for-age *z*-scores (HAZs). Second, we will examine the impact of province-level characteristics (i.e. demographic, economic and local government health spending) on the variation in health achievement in children.

The paper is structured as follows: the next section will describe the methodology, data, and variables. Section 3 reports the empirical results. A discussion of our study findings is reported in Section 4, and Section 5 provides a brief conclusion.

## Methods

### Methodology

This section summarizes two common measures of inequality: the concentration index; and health achievement. We follow this opening discussion with an outline of the empirical strategy proposed to examine province-level variations in health achievement over the last twenty years (1989–2009).

#### Measures of income-related health inequality and health achievement

The concentration index was first introduced to measure health inequality by Wagstaffet al. [24,25]. Building on the Lorenz and Gini coefficients, the concentration index is widely used for measuring income-related health inequality, and provides a means of qualifying the degree of income-related inequality in a specific health variable [7,18,22,26-28]. Denote *h*_*i*_ as ill health indicator for *i*^*th*^ child. In our study, ill health is the negative of the height-for-age *z*-score. N = {1, 2, 3…. *n*} as the sample size, *μ* as the sample mean of *h*, and *R*_*i*_ as the fractional rank in the living-standards distribution for individual *i*. Then the concentration index of *h*, denoted as *C*, is calculated as shown in equation (1). Within this framework, a measure of inequality in ill health is given by C. The value judgments implicit in C are seen most easily when it was rewritten in an equivalent way as shown in equation (2). The quantity (*h*_*i*_*/nμ*) is the share of ill health suffered by *i*^*th*^ child. This is then weighted in summation by twice the complement of the person’s fraction rank, that is, 2(1-*R*_*i*_). Thus, the poorest person’s ill health share is weighted by a number close to two. The range for C is from −1 to +1. The concentration index is frequently applied to measures of ill-health for which a negative concentration index indicates that ill-health (stunting) is more concentrated among children living in poorer families.

1 $$ C=\frac{2}{n\mu }{\displaystyle \sum_{i=1}^n{h}_i{R}_i}-1 $$

2 $$ C\kern0.5em =\kern0.5em 1\kern0.5em -\kern0.5em \frac{2}{n\mu }{\displaystyle \sum_{i=1}^n{h}_i\left(1\kern0.5em -\kern0.5em {R}_i\right)} $$

Wagstaff [29], in referencing Yizhaki’s [30] extended Gini coefficient, designed the extended concentration index (Equation 3). The weight attached to the *i*^th^ person’s health share (*h*_*i*_*/nμ*) is now equal to *v*(1-R_i_)^(v-1)^ where *v* represents the inequality-aversion parameter. When *v* = 1, everyone’s ill health is weighted equally. When *v* = 2, the weight is the same as in the regular concentration index; thus C(2) is the standard concentration index. The weights vary depending on the degree of inequality aversion (*v*). A higher level of *v* decreases the weight on higher wealth groups and increases the weight on the lower wealth groups. C(*v*) ranges between −1 and +1 and it takes negative values when ill-health outcomes are disproportionately concentrated among the poor.

3 $$ C(v)=\kern0.5em 1\kern0.5em -\kern0.5em \frac{v}{n\mu }{\displaystyle \sum_{i=1}^n{h}_i}{\left(1\kern0.5em -\kern0.5em {R}_i\right)}^{v-1} $$

The measure of “achievement” proposed by Wagstaff [29] combines the average level of health and inequality in health between the poor and the better-off. It is defined as a weighted average of the health levels of the various people in the sample, in which higher weights are attached to poorer people than to better-off people. Clarke and Hayes [31] compared changes in prevalence of various cardiovascular risk factors and absolute inequality in the distribution of these factors in Australia. They used the achievement plane which could track changes in health and inequality over time. Meheus and Van Doorslaer [32] analyzed trends of measles immunization coverage in the 1990s for 21 developing countries using the achievement index. Achievement can be measured by the index in equation (4) [29]. *I(v)* is equal to the equation (5), in which the mean level of ill-health is multiplied by one minus the extended concentration index. In our study, the health indicator is ill health (the negative of the HAZ) and C(*v*) < 0 (ill health is higher among the poor), a lower value of I(v) represents greater achievement.

4 $$ I(v)\kern0.5em =\kern0.5em \frac{1}{n}{\displaystyle \sum_{i=1}^n{h}_iv}{\left(1\kern0.5em -\kern0.5em {R}_i\right)}^{v-1} $$

5 $$ I(v)=\mu \left(1-C(v)\right) $$

#### Regression model specification

In order to examine province-level variation in health achievements in China, we modeled the health achievements of nine provinces from 1989 to 2009 with a fixed effect regression model. The model is formalized in equation (6).

6 $$ {\mathrm{Y}}_{\mathrm{j}\mathrm{t}}\kern0.5em =\kern0.5em {\upalpha}_{1\mathrm{j}\mathrm{t}}{\mathrm{X}}_{1\mathrm{j}\mathrm{t}}\kern0.5em +\kern0.5em {\upalpha}_{2\mathrm{j}\mathrm{t}}{\mathrm{X}}_{2\mathrm{j}\mathrm{t}}\kern0.5em +\kern0.5em {\upalpha}_{1\mathrm{j}\mathrm{t}}{\mathrm{X}}_{3\mathrm{j}\mathrm{t}}\kern0.5em +\kern0.5em {\updelta}_{\mathrm{j}}\kern0.5em +\kern0.5em {\upvarepsilon}_{\mathrm{j}\mathrm{t}} $$

Y_jt_ is health achievement for province *j* at year *t*; X_1jt_ is a vector of demographic variables; X_2jt_ is a vector of economic variables; X_3jt_ is a vector of local government health spending. δ_j_ is a time-invariant, provincial-specific error component. ε_jt_ is an time-varying error component, clustered at the province level. We modeled time-invariant, provincial unobserved heterogeneity as a fixed effect; that is, we allow δ_j_ to be correlated with X_jt_. The Hausman test (1978) is performed to test whether the fixed effect model is favored in this study. The null hypothesis for the Hausman test is no difference between coefficients estimated in the fixed effect and random effect models.

### Data

We used the CHNS data with eight waves in 1989, 1991, 1993, 1997, 2000, 2004, 2006, and 2009. In this period, the Chinese economy moved from a central planning system towards a market model. Food rationing at controlling price and subsidized food coupons disappeared in the early 1990s, simultaneously money income became more important to obtain food, and therefore more important for child health [33]. This study selected this period (1989–2009) to evaluate income-related health inequality and health achievement in China. The CHNS was a longitudinal survey collected by the Carolina Population Center at the University of North Carolina at Chapel Hill, the Institute of Nutrition and Food Hygiene, and the Chinese Academy of Preventive Medicine. The CHNS employed a random cluster sampling procedure to draw the samples from nine provinces in China, comprising Liaoning, Heilongjiang, Jiangsu, Shandong, Henan, Hubei, Hunan, Guangxi and Guizhou. In the first three waves (1989, 1991, and 1993), Heilongjiang Province was not included. In the 1997 wave, Liaoning Province was excluded. Although the sampling frame for the CHNS does not comprise the whole country, it is broadly representative of the diverse regions of China. Moreover, the response rate was high (averaging 88%) for each wave of the CHNS [34]. The Child questionnaire of CHNS is completed for all children under age 18 by themselves, but for children less than 10 years of age a parent answers the questions on behalf of the child. Persons age 18 and older complete the adult questionnaire. The sample for this study comprises children aged 0 to 17.99 years. Starting with a sample of 20,486 child respondents, after excluding missing data, the final study sample comprised 19,801 observations. The macro-level indicators were obtained from China Statistical Yearbook [35].

### Variables

Height-for-age *z*-scores (HAZs) have been wildly used as measures for children health across countries and for children with different background within countries [7,17,18,23,33]. The *z*-scores are calculated as the difference between actual height and mean height divided by the standard deviation in the reference children population of same age and gender. We used the reference standards of the World Health Organization’s (WHO) growth chart to compute HAZ. Generally, children whose HAZ is below minus two standard deviations of the median of the reference population are considered stunted.

Per capita household net income is regarded as an indicator to measure social and economic status. The CHNS collected detailed information on various income sources for households, including income from wages, agriculture, own business and public/private transfers. Household net income has been expressed in 2009 Yuan prices (USD1.00 = CNY 6.824) using consumer price indices specific to each wave, province and country and divided by the household size.

There are three sets of province characteristics used to explain the variation in health achievement. Xu [27] and E Xie [36] identified the association between child health and economic factors, demographic factors and local government health spending. In our study, demographics indicators included the proportion of the population aged 14 and below, primary school enrollments, middle school enrollments, and the urbanization rate. The definition of the urbanization rate is the proportion of the urban population in total population. Economic indicators included inflation-adjusted per capita gross domestic product at 2009’s constant price, the Engel’s coefficient and the unemployment rate. The Engel's coefficient is the proportion of income spent on food. The unemployment rate is defined as the proportion of the official registered unemployment population in the total working population. Province government spending indicators included per capita public health spending at 2009’s constant price, the number of health care institutions per 1,000 populations and hospital beds per 100,000 populations. Health care institutions include hospitals, primary care institutions, public health institutions, and other health care institutions.

## Results

### Descriptive results

**Figure 1:**
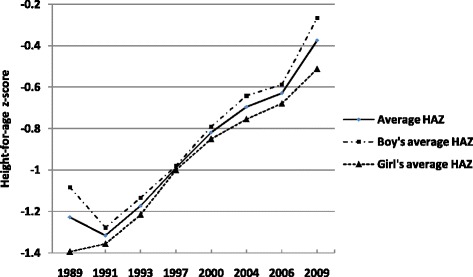
**Time trends of child health and health disparities for boys and girls.**

**Figure 2:**
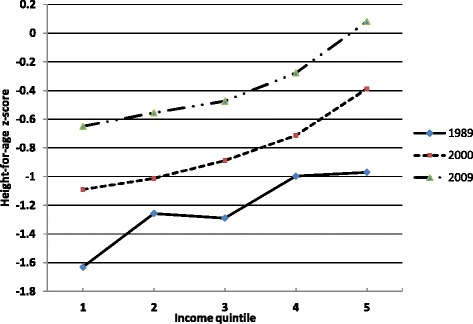
**Child health (Height-for-age** ***z*** **-score) by equivalent income quintile.**

Table 1 reports Chinese child HAZs for different income quintile over the period 1989 to 2009. The first column reports the mean of the HAZs. The increasing trend, from −1.227 in 1989 to −0.375 in 2009, suggests improvements in health status. Specially, the HAZ among the richest quintile was 0.08 in 2009 in the second to last column. This result suggests that the health status of children from households in the highest income group was better than the reference WHO standard. Figure 1 shows the trend of health disparities for boys and girls separately from 1989 to 2009. The raw general gap in HAZs persisted during this period. Boys had higher HAZs than girls. Table 1 shows that children’s health is generally better for families in higher income quintiles. For example, the mean HAZ is −1.34 when family income is in the bottom quintile (the poorest group), and −0.473 when it is in the top (the richest group). The HAZ for each income quintiles was shown in Figure 2. It had a positive income gradient, indicating that a greater share of health status was distributed among higher income families.

**Table 1:** **Chinese child HAZs for different income quintile over the period 1989 to 2009**

**Year**	**Average HAZ**	**Absolute Coefficient of Variation**	**Mean value of HAZs under different income quintiles**					**Sample Size**
			**1 (the poorest)**	**2**	**3**	**4**	**5 (the richest)**	
	**(1)**	**(2)**	**(3)**	**(4)**	**(5)**	**(6)**	**(7)**	**(8)**
1989	−1.227	1.136	−1.631	−1.257	−1.288	−0.996	−0.970	1605
1991	−1.316	0.873	−1.611	−1.521	−1.362	−1.100	−0.987	3883
1993	−1.172	0.991	−1.428	−1.399	−1.185	−0.998	−0.854	3443
1997	−0.989	1.187	−1.227	−1.221	−1.056	−0.796	−0.648	2973
2000	−0.818	1.436	−1.091	−1.014	−0.888	−0.713	−0.388	2762
2004	−0.695	1.853	−1.062	−0.902	−0.698	−0.549	−0.272	1939
2006	−0.630	2.111	−0.867	−0.846	−0.640	−0.511	−0.277	1614
2009	−0.375	3.664	−0.649	−0.556	−0.475	−0.279	0.080	1582
Total	−0.973	1.297	−1.340	−1.239	−1.033	−0.782	−0.473	19801

*Note*: Authors’ calculations from the CHNS data (1989, 19991, 1993, 2000, 2004, 2006 and 2009).

Table 2 presents descriptive statistics for the study variables. The mean proportion of the population aged 14 and younger was 23% in all samples from 1989 to 2009. The mean value of primary and middle school enrollments were 5.76 and 3.19 million, respectively. China has been experienced rapid urbanization, leading to a rise in the urban population from 191 million in 1980 to 622 million in 2009 [37]. The mean urbanization rate was 0.28 in our sample. There was wide variation in inflation-adjusted per capita Gross Domestic Product (GDP) across Chinese provinces with more than a 28-fold difference between the richest and poorest provinces from 1989 to 2009. The mean value of per capita GDP was 10,116.88 YUAN. The mean unemployment rate was 3.4%, but the maximum value was 6.3%. The unemployed are captured in Official statistics as only those individuals who register for unemployment benefits with local governments. The Engel’s Coefficient fell from 0.55 in 1989 to 0.40 in 2009. Over the study period, the mean public health spending per capita was 87.27 YUAN. It has increased rapidly over the last two decades. Specifically, the public health spending per capita increased from 17.61 YUAN in 1989 to 269.7 YUAN in 2009. Over this period, the average annual rate of growth was 56.57%. The mean health care institutional density per 1,000 people was 14.5, and hospital beds per 10,000 were 15.6.

**Table 2:** **Variables definition and the data descriptive statistics**

**Variable**	**Definition**	**mean**	**Sd.**	**min**	**max**
Age14	Population Proportion aged 14 and below (%)	0.230	0.056	0.110	0.340
Primary school enrollments	(million)	5.762	2.304	1.904	11.70
Middle school enrollments	(million)	3.194	1.560	0.933	7.594
Urbanization rate	Proportion of urban population (%)	0.276	0.126	0.120	0.500
GDP	Inflation-adjusted per capita gross domestic product (RMB) at 2009’s constant price	10116.88	9167.58	1558	44744
Engel coefficient	Proportion of income that goes into food	0.482	0.076	0.334	0.638
Unemployment rate	Proportion of official registered unemployment population in the total working population	0.034	0.010	0.016	0.063
Health expenditure	Per capita public health spending (RMB) at 2009’s constant price	87.273	92.191	16.891	377.19
Health institutions density	Health care institutions density per 1,000 population	14.463	12.498	3.908	75.722
Hospital beds	Hospital beds per 10,000 population	15.613	5.792	5.70	34.70

*Note*: Authors’ calculations from the CHNS data (1989, 19991, 1993, 2000, 2004, 2006 and 2009).

### Concentration index and health achievement

**Figure 3:**
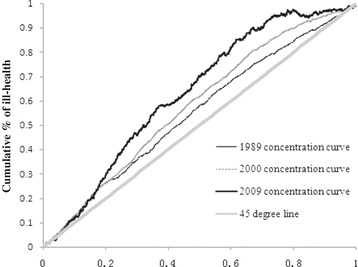
**Concentration curves ranked by income.**

To gain further insight into income-related health inequality in children, Table 3 presented the concentration index and health achievement. First, consider columns (1)-(4) for the concentration index and the extended concentration index. Without any exception, the concentration index C(2), see column (1), was negative. The results indicated that the poor had a greater share of ill-health in China, which we refer to as ‘pro-rich’ inequality. Moreover, C(2) had substantial between–year variation, ranging from −0.099 in 1989 to −0.366 in 2009. Figure 3 presented the concentration curves of 1989, 2000, and 2009. The concentration curve was derived by ranking per capita household net income on the *x*-axis and plotting the cumulative proportion of ill-health on the *y*-axis. In the case of complete equality the concentration curve would lie on this diagonal and the value of the concentration index would be zero. In Figure 3, three concentration curves lie above the diagonal. The curve for 2009 was higher than the other two. Figure 3 suggests that ill health became more concentrated among the relatively poor over the last two decades. In Table 3, four degrees of inequality aversion were used from *v* = 2, 3, 4 and 5. As we mentioned before, raising the value of *v* above 2, the weight attached to the health of the poor increases. For example, the value of C(2) was only −0.366 whilst the value of C(5) was −0.569 in 2009. This result implies that increasing health inequality was found in the poor families. We took account of health inequality as well as the average level of health in the assessment of health achievement. In Table 3 consider columns (5) for health achievement. When we tracked changes over time, it showed that health achievement improved from 1.35 in 1989 to 0.514 in 2009. As expected, raising the inequality-aversion parameter *v* from 2 to 5 resulted in an increase in the health achievement indices, meaning that the level of “disachievement” became larger and larger as seen by moving from column (5) to column (8).

**Table 3:** **Concentration index, extended concentration index and health achievement of children**

		**C(** ***v*** **)**					**I(** ***v*** **)**	
**Year**	**(1)**	**(2)**	**(3)**	**(4)**	**(5)**	**(6)**	**(7)**	**(8)**
	***v*** **= 2**	***v*** **= 3**	***v*** **= 4**	***v*** **= 5**	***v*** **= 2**	***v*** **= 3**	***v*** **= 4**	***v*** **= 5**
1989	−0.099	−0.150	−0.179	−0.196	1.350	1.412	1.446	1.468
1991	−0.104	−0.152	−0.178	−0.192	1.453	1.516	1.550	1.569
1993	−0.109	−0.157	−0.180	−0.191	1.301	1.356	1.383	1.396
1997	−0.127	−0.180	−0.200	−0.206	1.115	1.1670	1.187	1.193
2000	−0.172	−0.237	−0.265	−0.2780	0.959	1.012	1.035	1.046
2004	−0.234	−0.344	−0.410	−0.454	0.858	0.934	0.980	1.011
2006	−0.193	−0.272	−0.309	−0.328	0.752	0.801	0.824	0.836
2009	−0.366	−0.504	−0.555	−0.569	0.514	0.565	0.583	0.588

*Note*: Authors’ calculations from the CHNS data (1989, 19991, 1993, 2000, 2004, 2006 and 2009).

### Regression results of province-level health achievement

Table 4 reported the fixed effects regression of province-level characteristics associated with health achievement when inequality-aversion parameter was equal to 2. Demographics, economic indicators and provincial government health spending explained more than 95% of the variation in province-level health achievement, as indicated by the regression R^2^. Among demographic characteristics, more middle school enrollments were significantly associated with higher health achievement. Among economic indicators, higher GDP was significantly associated with health achievement (P = 0.049). Provinces with a higher proportion of urbanites had significantly greater health achievement (P < 0.0001). Among provincial government health spending indicators, and as expected, per capita public health spending was positively associated with health achievement (P < 0.0001). We controlled for time-invariant provincial characteristics heterogeneity via fixed effects.

**Table 4:** **Fixed effects regressions of province-level characteristics with I (2)**

**Variable**	**Coefficient**	**Std. Err.**	**t-value**	**p-value**
Age14	−1.974†	1.094	−1.80	0.081
Primary school enrollments	−0.0001	0.0001	0.12	0.906
Middle school enrollments	−0.0004*	0.0002	−2.62	0.048
Urbanization rate	−2.729***	0.606	−4.51	0.000
LnGDP	−0.236*	0.116	−2.04	0.049
Engel Coefficient	0.838	0.681	1.23	0.228
Unemployment rate	0.458	2.455	−0.19	0.853
Health expenditure	−0.002***	0.0003	−5.86	0.000
Health Institutions Density	0.005	0.003	1.53	0.136
Hospital beds	0.026	0.017	1.35	0.106
Constant	3.567**	1.221	2.92	0.006
R^2^	0.9505			
F-test	F (10, 31) =59.59***			0.0000
Hausman-test	chi2 (7) =27.96***			0.0002

*Note*: Authors’ calculations from the CHNS data (1989, 19991, 1993, 2000, 2004, 2006 and 2009).

†P < 0.1, *P < 0.05, **P < 0.01, ***P < 0.001.

### Sensitivity analysis

As we mentioned, when the inequality-aversion parameter is equal to 2 (*v* = 2), the extended concentration index reverts to the standard concentration index. We looked at the I(2) as our base case. Table 5 reports province-level characteristics associated with I(3), I(4) and I(5) to examine how sensitive the results are to alternative values to *v*. Our results were very similar to the base case analysis. Health achievement was positively impacted by middle school enrollments, the urbanization rate, inflation-adjusted per capita gross domestic product, and per capita public health spending. These findings suggest that our results were quite robust to the value of *v* selected. Only the health care institutional density variable had different regression results. Rising with *v*, the health care institutional density had a negative impact on province-level health achievement.

**Table 5:** **Fixed effects regressions of province-level characteristics with I(3), I(4), and I(5)**

	**I(3)**		**I(4)**		**I(5)**	
**Variable**	**Coef.**	***Sig.***	**Coef.**	***Sig.***	**Coef.**	***Sig.***
Age14	−2.134	†	−2.225	†	−2.248	
Primary school enrollments	−0.0001		−0.0001		−0.0001	
Middle school enrollments	−0.0005	*	−0.0005	*	−0.0005	†
Urbanization rate	−3.04	***	−3.30	***	−3.497	***
LnGDP	−0.239	†	−0.243	†	−0.244	†
Engel Coefficient	0.946		0.966		0.933	
Unemployment rate	0.588		1.497		2.172	
Health expenditure	−0.0022	***	−0.0023	***	−0.0023	***
Health Institutions Density	0.007	†	0.008	*	0.0086	†
Hospital beds	0.020		0.166		0.013	
Constant	3.777	**	3.948	**	4.082	**
R^2^	0.9525		0.9488		0.9426	
Hausman-test	chi2 (7) =27.71	***	chi2 (7) =26.82	***	chi2 (7) =25.67	***

*Note*: Authors’ calculations from the CHNS data (1989, 19991, 1993, 2000, 2004, 2006 and 2009).

†P < 0.1, *P < 0.05, **P < 0.01, ***P < 0.001.

The base case analysis of the impact of province-level characteristics on health achievement in children is reflective to two influences: mean health and health inequality. It is difficult for an outcome to fully capture different influence pathways on health and health inequality. So we additionally investigated these two outcomes separately in Table 6. We found that inflation-adjusted per capita gross domestic product simultaneously improved mean health and increased health inequality. However, the combined effect resulted in an improvement in health achievement. It was shown that per capita public health spending not only improved mean health for children but also reduced inequality at the provincial level. We found a significant positive relationship between urbanization and children’s health. At a marginal level of significance, the Engel Coefficient was negatively associated with the concentration index.

**Table 6:** **Fixed effects regressions of province-level characteristics with health and health inequality**

	**I(2)**		**Health**		**C(2)**	
**Variable**	**Coef.**	***Sig.***	**Coef.**	***Sig.***	**Coef.**	***Sig.***
Age14	−1.974	†	1.940		−0.505	
Primary school enrollments	−0.0001		−0.0001		−0.0003	
Middle school enrollments	−0.0004	*	0.0003		−0.0002	
Urbanization rate	−2.729	***	2.735	***	0.609	
LnGDP	−0.236	*	0.261	*	−0.365	†
Engel Coefficient	0.838		−0.465		−2.006	†
Unemployment rate	0.458		0.027		−1.375	
Health expenditure	−0.0021	***	0.0017	***	0.0016	**
Health Institutions Density	0.005		0.0007		−0.013	*
Hospital beds	0.026		−0.044		0.057	
Constant	3.567	**	3.547	*	3.174	
R^2^	0.9505		0.9488		0.4435	
Hausman-test	chi2(7) =27.96	***	chi2(7) =26.52	***	chi2(7) =28.97	***

*Note*: Authors’ calculations from the CHNS data (1989, 19991, 1993, 2000, 2004, 2006 and 2009).

†P < 0.1, *P < 0.05, **P < 0.01, ***P < 0.001.

We ran a sensitivity analysis, fixed effects regressions of province-level characteristics with I(2), separately for children below and beyond 10 years of age in Table 7, in order to check whether the results are dependent on whether the parents or the children themselves completed the questionnaire. We found the results from that the sample of children below 10 years of age was similar to the pooled sample.

**Table 7:** **Fixed effects regressions of province-level characteristics with I(2) for children below and beyond 10 years of age**

	**Pooled Sample**		**Below 10 years of age**		**Beyond 10 years of age**	
**Variable**	**Coef.**	***Sig.***	**Coef.**	***Sig.***	**Coef.**	***Sig.***
Age14	−1.974	†	−4.296	*	0.797	
Primary school enrollments	−0.0001		−8.34e-5		−2.38e-6	
Middle school enrollments	−0.0004	*	−0.0007	†	2.3e-4	***
Urbanization rate	−2.729	***	−3.36	**	−2.051	*
LnGDP	−0.236	*	−0.4208	*	0.067	
Engel Coefficient	0.838		0.6873		1.741	
Unemployment rate	0.458		1.0450		−4.558	
Health expenditure	−0.002	***	−0.0021	**	−0.0020	***
Health Institutions Density	0.005		0.0037		0.0077	†
Hospital beds	0.026		0.055	†	−0.0105	
Constant	3.567	**	5.462	*	0.282	
R^2^	0.9505		0.8611		0.9171	
Hausman-test	chi2(7) =27.96	***	chi2(7) =21.51	**	chi2(7) =20.56	**

*Note*: Authors’ calculations from the CHNS data (1989, 19991, 1993, 2000, 2004, 2006 and 2009).

†P < 0.1, *P < 0.05, **P < 0.01, ***P < 0.001.

## Discussion

The present study showed that the average health status as measured by HAZs for children improved in the two decades following 1989. Our results were consistent with earlier studies [21-23,33]. There are two potential explanations for the improvement in the health status of children. First, China’s economic reforms have raised living standards. Food diversity and high protein food consumption increased considerably in both urban and rural China. Second, there have been significant improvements to China’s health system that has enhanced access to preventive health services, especially for child and maternal care. However, we found good health is significantly concentrated among the higher end of the income distribution. A recent article by Goode et al. [38] found significant child health/family income gradients among Chinese children aged between 3 and 17, and these gradients strengthen with age until the age of 12.

Our study findings regarding the negative concentration index suggests that the poor had a greater share of ill-health in China, i.e. there was ‘pro-rich’ health inequality. An increase in the inequality-aversion parameter, the weight attached to the health of a poor person, resulted in an increase in inequality. Moreover, inequality widened over the last two decades. The findings of the current study are consistent with previous research on income-related children’s health inequality in developing countries, such as Vietnam, Nigerian, and India. Wagstaff et al. [17] used data from the 1993 and 1998 Vietnam Living Standard survey (VLSS) and found inequalities in stunting (low height-for-age *z*-scores) amongst young children were due largely to inequalities in household consumption and to inequalities in unobserved determinants at the commune level. In Uthman’s [18] study of childhood malnutrition in Nigeria, he found the concentration index, indicating socioeconomic inequality of under-five childhood malnutrition, was −0.147. This concentration index suggested that there were significant differences in under-five child malnutrition that favored the better-off in Nigerian society. Arokiasamy and Pradhan [19] found that below two years of age deaths have been significantly concentrated among the poor households in India.

The health achievement indicator allows us to capture both efficiency (i.e. average health) and equity (i.e. income-related inequality) considerations. Although children’s health achievement in China has been improving over time, the pro-rich inequality component has lessened the associated gain in achievement. The larger the inequality-aversion parameter, the stronger the negative impact on health achievement. Although equity is an important goal of health policy, it is not the only goal. From the perspective of policy decision makers, they are likely to be willing to trade off increases in inequality against improvements in the average level of health [29].

In our study, we found that there were substantial variations in health achievement across provinces in China. Provinces with better health achievement had greater enrollments in middle school. This suggests that health achievement tends to increase when provincial governments allocate more resources to education. Provincial health achievement was positively associated with urbanization. Our result is consistent with a recent article by Gong et al. [37]. Gong et al. found that health benefits continued to accrue to urban populations, who have better access to health services, education and higher incomes than their rural counterparts. The Growth of GDP is a double-edged sword as it tends to improve mean health status, but also aggravates health inequality. In our analysis, the combined effect of the growth of GDP was in the direction of improvements in health achievement. The negative correlation between per capita public health spending and health achievement is further strengthened by the finding that provincial government spending on public health significantly increased health achievement for children. However, we found that greater health care institutional density was associated with a worsening of health inequality, particularly rising with the inequality-aversion parameter. The potential explanation was greater take-up of health care services among higher income groups. Song [39] estimated income-related inequalities in health care use and found community health and medical services were actually widening inequalities in the health of children instead of providing protection.

Several limitations should be acknowledged. First, due to data limitations, we only used HAZs to measure child health. But stunting is only one of many important indicators of child health status. The CHNS data did not provide detailed child health indicators, such as under-2 child mortality. Second, our data set involved nine Chinese provinces, representing 42% of total population of China [35]. However, CHNS data provided us with a good opportunity to conduct a 20-year’s study on trends in child health status and health inequality in China, and captures regions that are broadly representative of the whole country. Third, macro-level indicators used in our analysis were imperfect as some data were not available. Specifically, the provincial statistical office of China did not publish children-related macro data, such as children-related health care institutions and health expenditures just for children. Forth, although it would have been better if we had been able to use the percentage of school-age children enrolled and the percentage of graduates of primary schools entering middle schools, rather than the level of enrollment, only four years of data were available from all nine sample provinces. In order to retain all eight sample years, we used only educational variables in absolute numbers.

## Conclusion

This study has provided evidence that average health status of Chinese children has improved from 1989 to 2009, but inequality widened. In other words: widening inequality slowed the growth in health achievement for children over time. Our results should be considered in the context of current and proposed Chinese government policy decision-making. It is clear that one way to improve health achievement is by increasing the mean level of health, while simultaneously reducing inequality. Although policy makers may trade-off improvements in equality against improvements in the mean of the distribution, they should take into account how far society is prepared to accept greater health inequality in order to achieve greater mean health status. We found variation across provinces in the magnitude of health achievement. Our study established that health achievement was positively associated with the distribution of income as well as improvements in education, urbanization, and medical and public resources. Future research should empirically test the conjecture that increases in income, education, and urbanization, as well as prioritizing efforts to reallocate medical and public health resources indeed lead to better health achievement in children.

### Consent

Written informed consent was obtained from the patient’s guardian/parent/next of kin for the publication of this report and any accompanying images.

## Abbreviations

CHNS: China Nutrition and Health Survey
HAZs: Height-for-age *z*-scores
WHO: World Health Organization
GDP: Gross Domestic Product
